# Sequential repeated tibial tubercle osteotomy in a two-stage exchange strategy: a superior approach to treating a chronically infected knee arthroplasty?

**DOI:** 10.1007/s00590-023-03548-4

**Published:** 2023-04-20

**Authors:** Pablo S. Corona, Marta Pérez, Matías Vicente, Oriol Pujol, Carles Amat, Lluís Carrera

**Affiliations:** 1 Universitat Autònoma de Barcelona (UAB), Orthopaedic Surgery Department, Vall d’Hebron University Hospital, Barcelona, Spain; 2 Septic and Reconstructive Surgery Unit (UCSO), Orthopaedic Surgery Department, Vall d’Hebron University Hospital, Universitat Autònoma de Barcelona (UAB), Barcelona, Spain; 3 Musculoskeletal Tissue Engineering Group, Vall d’Hebron Research Institute, Barcelona, Spain; 4 Surgery Department, Universitat Autònoma de Barcelona (UAB), Barcelona, Spain

**Keywords:** Tibial tubercle osteotomy, Periprosthetic joint infection, Two-stage revision arthroplasty, Infection control

## Abstract

**Purpose:**

Surgical approach can impact the reliability of the debridement after a chronic total knee periprosthetic joint infection (PJI), a factor of utmost importance to eradicate the infection. The most adequate knee surgical approach in cases of PJI is a matter of debate. The purpose of this study was to determine the influence of performing a tibial tubercle osteotomy (TTO) in a two-stage exchange protocol for knee PJI treatment.

**Methods:**

Retrospective cohort study examining patients managed with two-stage arthroplasty due to chronic knee PJI (2010–2019). Performance and timing of the TTO were collected. Primary end-point was infection control with a minimum FU of 12 months and according to internationally accepted criteria. Correlation between TTO timing and reinfection rate was reviewed.

**Results:**

Fifty-two cases were finally included. Overall success (average follow-up: 46.2 months) was 90.4%. Treatment success was significantly higher among cases addressed using TTO during the second stage (97.1% vs. 76.5%, *p* *value* 0.03). Only 4.8% of the patients relapsed after performing a sequential repeated TTO, that is, during both first and second stages, compared to 23.1% cases in which TTO was not done (*p* value 0.28). No complications were observed among patients in the TTO group with a significant decrease in soft tissue necrosis (*p*: 0.052).

**Conclusion:**

Sequential repeated tibial tubercle osteotomy during a two-stage strategy is a reasonable option and offers high rates of infection control in complex cases of knee PJI with a low rate of complications.

## Introduction

Periprosthetic joint infection (PJI) is one of the most devastating complications following total knee arthroplasty (TKA) and one of the most challenging problems an orthopedic surgeon faces [1].

Although direct exchange arthroplasty has been proven a reliable strategy in certain scenarios [2], two-stage reconstruction has been the widely accepted model of care [3]. However, a superior two-stage treatment algorithm is still lacking, and management of chronic knee PJI remains controversial [4, 5].

Surgical debridement quality is of utmost importance in all surgical protocols to eradicate infection [6]. Surgical approach can impact the reliability of the surgical debridement. Several approaches have been proposed, including an extensile medial parapatellar (EMP) approach and tibial tubercle osteotomy (TTO) [7, 8]. The latter approach likely allows better access, especially to the knee’s difficult-to-reach lateral aspect, thus facilitating more complete debridement [9–11]. However, the influence of this approach on infection control rates after a two-stage strategy has been poorly studied, as has the rate of TTO complications in the infected scenario [9, 12, 13].

Considering all factors discussed above, we sought to analyse the influence of the surgical approach in a consecutive series of chronic knee PJI managed with a two-stage exchange strategy and to study any association between performance of a tibial tubercle osteotomy and: (1) risk of overall infection treatment failure; (2) incidence of TTO-related complications; and (3) association of TTO-timing with final outcomes.

Our primary hypothesis is that the use of a sequential repeated TTO in a two-stage strategy after chronic PJI achieves better infection control rate than in cases where an osteotomy is not performed, without increasing complication rate.

## Materials and methods

### Study design

After Institutional Review Board (IRB) approval (date: 13/11/2020, reference number: PR(ATR)283/2020), we conducted a retrospective review to identify all consecutive chronic knee PJIs managed with two-stage revision from January 2010 through December 2019. In order to homogenize the sample only cases with a single manufacturer's implant system used during the second stage were accepted. In this series, a cemented modular rotational hinge revision arthroplasty (CMRH) was used in all cases during the second stage.

### Inclusion–exclusion criteria

Two-stage strategy to manage chronic knee PJI. Use of the same CMRH implant during the second stage. Minimum follow-up of 12 months after the second stage. All included patients had an established diagnosis of chronic PJI according to an internationally accepted definition [14]. Chronic PJI was defined as any PJI present more than four weeks from the index procedure [15]. Patients who did not fit all inclusion criteria and those treated with a distal femur megaprosthesis during reimplantation were excluded from the study.

### Outcome variables

Primary end-point was infection control rate. Patient demographic variables, American Society of Anesthesiologists (ASA) Scale, Charlson’s Comorbidity Index (CCI) [16] and McPherson’s host classification [17] were collected, as were the type of infected prosthesis and number of previous surgeries.

First-stage and second-stage-related variables; date of surgical procedure, TTO performance, type of spacer (static/dynamic), final modular reconstruction, microorganisms and soft tissue reconstructive procedures were reviewed. Spacer-stage variables: spacer-related complications, reoperations and TTO-related complications. Post-operative data included TTO-related complications, need for unexpected reinterventions, infection relapse.

The patients were divided into three groups according to whether tibial tubercle osteotomy had been performed, and the timing of TTO; Group A: repeated sequential TTO (during both first and second stages); Group B: single TTO (in either first or second stage) and Group C: no TTO. Osteotomy was considered healed when radiographic evidence of bridging callus formation was observed on lateral radiography.

### Two-stage operative technique

Members of our centre’s Septic Unit (three surgeons) performed all operations. In the first stage, the earlier prosthesis and cement were removed. An EMP approach (including neither quadriceps snip nor quadriceps turndown) or TTO was performed; the latter being chosen when a safe mobilization of the extensor mechanism could not be achieved by an EMP approach or when correct visualization of the knee was compromised due to stiffness and rigidity. Thorough debridement and irrigation were performed. At least six solid samples were obtained for microbiological culture, as well as tissue samples for histological examination.

**Fig. 1:**
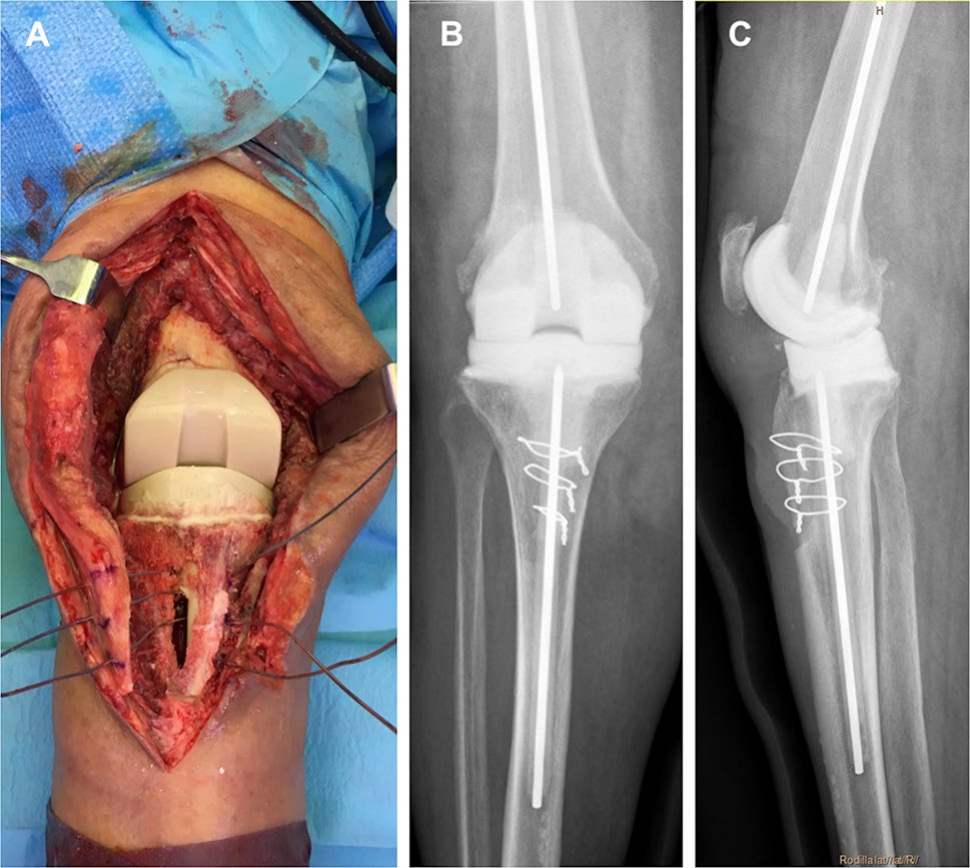
**a** First stage tibial tubercle osteotomy (TTO) approach. The cerclages pass behind the spacer stem; **b** anteroposterior and **c** lateral radiography of a prefabricated mobile spacer

The TTO technique (Fig. 1a) is based on the technique described by Whiteside in 1995 [11] and which can be found elsewhere; osteotomy is normally secured with three wires cerclages, passed behind the tibial stem.

**Fig. 2:**
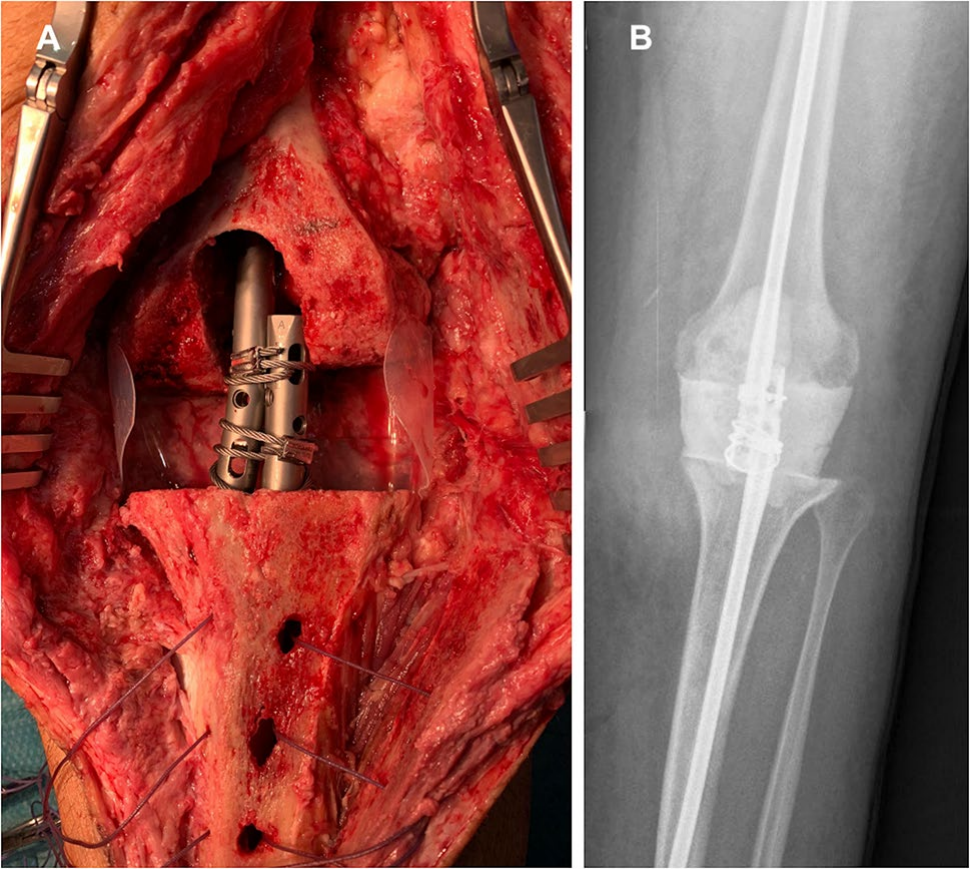
**a** First stage tibial tubercle osteotomy (TTO) approach with a static spacer; **b** anteroposterior radiography of the static spacer

In the first stage, our technique of choice includes use of a mobile prefabricated knee spacer. In such cases, a vancomycin-gentamicin prefabricated antibiotic-cement spacer (*Vancogenx®*, Tecres SpA, Sommacampagna, Verona, Italy) is used, fixed with a vancomycin-gentamicin-loaded acrylic bone cement (*Vancogenx®* bone cement, Tecres SpA, Sommacampagna, Verona, Italy) with an extra dose of powder antibiotic [4]. Usually, a hand-made antibiotic-loaded cement stem (reinforced with a Steinmann pin) is connected to the spacers to increase stability and fill the intramedullary dead space (Fig. 1b, c). In cases of TTO, the stem is mandatory and must bypass the osteotomy by a minimum of 5 cm to avoid fracture of the tibia [18, 19]. In cases of knee infection with massive bone defects, extensor mechanism disruption, or soft tissue deficiencies, we prefer to use a static spacer [20] (Fig. 2a, b).

All patients followed similar post-operative antibiotic protocols, as recommended by an infectious-diseases expert [21], member of our specialty-dedicated unit. In general, the antibiotic treatment was selected according to the susceptibility profile of the bacteria present, and following the clinical practice guidelines of the Spanish Society of Infectious Diseases and Clinical Microbiology (SEIMC). Regarding systemic antibiotics, treatment was initiated with intravenous antibiotics for 8–10 days, in which we usually used a beta-lactam, or a carbapenem if involvement of multi-resistant microorganism was suspected, associated or not with a glycopeptide or a lipopeptide. When final microbiological data and proper wound healing was confirmed, antibiotics were switched to oral and maintained for at least 6 weeks. Whenever possible, a combination of rifampicin with a second antibiotic was used for gram-positive infection. If susceptible, the preferred combination was rifampicin plus levofloxacin in the case of staphylococcal infection. If the selected antibiotic was linezolid, rifampicin was not added, due to the increased metabolism of linezolid which can result in decreased serum levels. In gram-negative infections, whenever susceptible, oral ciprofloxacin was administered. After cessation of antibiotic treatment, a minimum two-week antibiotic vacation period was begun. Timing of reimplantation was based on clinical improvements and laboratory values.

**Fig. 3:**
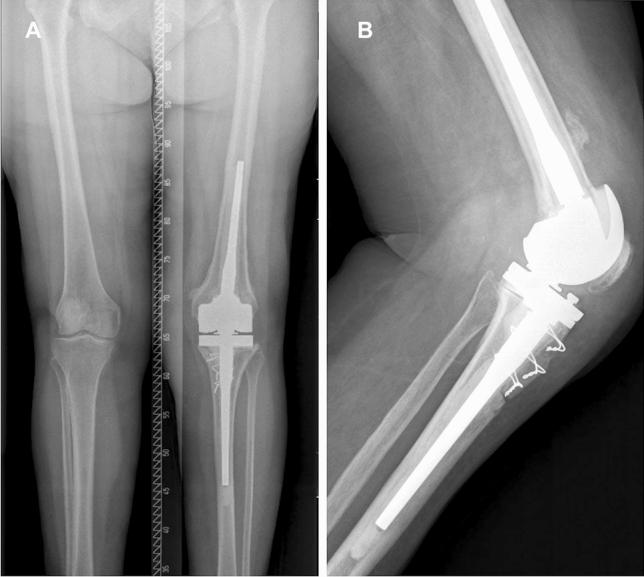
**a** Anteroposterior and **b** lateral post-operative radiography showing an Endo-Model®-M knee revision prosthesis

In the second stage, the spacer was removed, a second aggressive debridement was performed, and samples were collected. Either an EMP approach or a TTO was performed. Joint reconstruction was performed by implantation of a single design of CMRH prosthesis (Endo-Model®-M, Waldemar Link GmbH&Co.®; Hamburg; Germany), fixed with *Vancogenx®* bone cement (Fig. 3a, b). After both first and second stages, the patient is at rest without flexing the knee until correct evolution of the surgical incision is verified (this is usually not earlier than 10–14 days).

Following operation, systemic antibiotics against the first-stage-isolated microorganism were administered until availability of microbiological results. If, after seven to ten days, cultures were deemed negative, antibiotic treatment was withdrawn [4].

Success of the two-stage exchange protocol was defined as infection control according to an internationally accepted definition [22]: (a) healed wound without fistula or drainage and no infection recurrence caused by the same organism strain; (b) no subsequent surgical intervention for infection after reimplantation surgery; and (c) no PJI-related mortality. In addition, need for suppressive antibiotic treatment or the onset of another PJI caused by a different microorganism were also considered failure criteria. If one or more of the stated criteria was fulfilled, treatment failure was considered as established [4].

### Statistical analysis

Demographic-clinical characteristics were summarized as counts and percentages for categorical variables. Means and confidence intervals were calculated for continuous variables. Normality was tested using the Kolmogorov–Smirnov test. Groups were compared using the Chi-square or Fisher exact test (analysis of small samples) for categorical variables. Continuous variables were evaluated with the Student *t* test and ANOVA test (normal-distribution data), and the Wilcoxon–Mann–Whitney test and Kruskal–Wallis test (non-normal data). All *p* values were two-tailed; *p* values < 0.05 were considered statistically significant. A Kaplan–Meier estimate was conducted for any variable identified as a factor for better outcomes. Differences in the curves were evaluated with the Tarone–Ware test. *R* software was used to perform the aforementioned tests (*R* Core Team, 2020. *R* Foundation for Statistical Computing, Vienna, Austria).

## Results

**Fig. 4:**
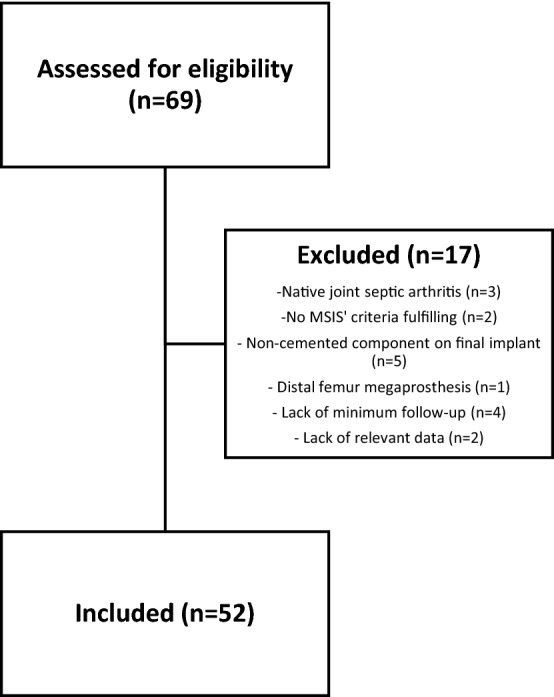
Flowchart of study cases. MSIS, Musculoskeletal Infection Society

In our database review, we detected sixty-nine cases of two-stage revision knee arthroplasty. After exclusion of patients who did not meet selection criteria **(**Fig. 4**)**, fifty-two cases of chronic knee PJI who had undergone a two-stage exchange arthroplasty protocol were finally included. Of these, 53.8% were females; average patient age was 71.5 ± 8.2 years. Comorbidities were common among the study population: 61.5% (32/52) were classified ASA III, 50% (26/52) were McPherson Type B, and 25% (13/52) had 3 to 5 points on CCI. In addition, 19.2% (10/52) had a PJI on a revision prosthesis, and 11.5% (6/52) were relapsed cases following previous unsuccessful two-stage replacement attempts. Patient characteristics are detailed in Table 1.

**Table 1:** Demographics, comorbidities and infection characteristics of the 52 patients

Variables	All patients *N* = 52 (100%)	Success *N* = 47 (90.4%)	Failure *N* = 5 (9.6%)	*p*
Age, years; mean (IQR range)	71.5 (69.2–73.7)	71.7 (69.2–74.2)	69.2 (66.5–71.9)	0.121
Sex, male/female	24 (46.2)/28(53.8)	21 (44.7)/26(55.3)	3 (60)/2 (40)	0.352
Diabetes mellitus	17 (32.6)	15 (31.9)	2 (40)	1
Smoking	1 (1.9)	1 (2.1)	0	1
Obesity	10 (19.2)	8 (17)	2 (40)	0.242
Alcoholism	1 (1.9)	1 (2.1)	0	1
Malignant neoplasm	8 (15.4)	7 (14.9)	1 (20)	1
Arthritis	4 (7.7)	4 (8.5)	0	1
Cirrhosis	1 (1.9)	1 (2.1)	0	1
Coagulation problems	7 (13.5)	6 (12.8)	1 (20)	0.53
*Charlson comorbidity index*				
≤ 2	33 (63.5)	30 (63.8)	3 (60)	0.795
3–5	13 (25)	11 (23.4)	2 (40)	
6–8	5 (9.6)	5 (10.6)	0	
≥ 9	1 (1.9)	1 (2.1)	0	
*ASA scale*				
II	19 (36.5)	16 (34)	3 (60)	0.411
III	32 (61.5)	30 (63.8)	2 (40)	
IV	1 (1.9)	1 (2.1)	0	
*McPherson*				
Type A	22 (42.3)	20 (42.6)	2 (40)	1
Type B	26 (50)	23 (48.9)	3 (60)	
Type C	4 (7.7)	4 (8.5)	0	
*Infection scenario type*				
Revision prosthetic infection	10 (19.2)	9 (19.1)	1 (20)	0.879
Primary prosthetic infection	31 (59.6)	28 (59.6)	3 (60)	
Primary prosthesis with previous failed DAIR	5 (9.6)	5 (10.6)	0	
Relapse of previous replacement due to infection	6 (11.5)	5 (10.6)	1 (20)	
*Number of previous replacement due to infection failed attempts*				
No previous failed attempts	46 (88.5)	42 (89.4)	4 (80)	0.473
One previous failed attempt	6 (11.5)	5 (10.6)	1 (20)	
N surgeries previous to first time; mean (IQR range)	1.9 (1.6–2.3)	1.9 (1.5–2.3)	1.8 (0.8–2.8)	0.975

In this series, 88.5% (46/52) of the spacers implanted were dynamic; 11.5% (6/52) were static. In 56.5% (26/46) of the dynamic spacers, a hand-made stem was added (Table 2). Spacer dislocations were detected in two dynamic-spacer cases (3.8%); there were no spacer breakages. We found no dislocation or fracture among static spacers.

**Table 2:** Univariate analysis of all variables investigated as predictors of failure in two-stage reimplantation

Variables	All patients *N* = 52 (100%)	Success *N* = 47 (90.4%)	Failure *N* = 5 (9.6%)	*p*
*Spacer type*				
Premade Vancomycin-Gentamicin with stem	26 (50)	24 (51.1)	2 (40)	0.829
Premade Vancomycin-Gentamicin without stem	19 (36.5)	16 (34)	3 (60)	
Premade Gentamicin	1 (1.9)	1 (2.1)	0	
Self-made static spacer	6 (11.5)	6 (12.8)	0	
*PJI-causing microorganisms*				
Gram positive	27 (51.9)	24 (51.1)	3 (60)	0.402
Gram negative	11 (21.2)	11 (23.4)	0	
Anaerobe	10 (19.2)	10 (21.3)	0	
Fungus	0	0	0	
Polymicrobial	8 (15.4)	8 (17)	0	1
*First stage approach*				
EMP	27 (51.9)	24 (51.1)	3 (60)	1
TTO	25 (48.1)	23 (48.9)	2 (40)	
*Second stage approach*				
EMP	17 (32.7)	13 (27.7)	4 (80)	**0.034**
TTO	35 (67.3)	34 (72.3)	1 (20)	
*Type of failure*				
Infection relapse (same bacteria)	1 (1.9)	–	1 (20)	–
Superinfection (different bacteria)	4 (7.7)	–	4 (80)	–
Kept the prosthesis at the end of the follow up	48 (92.3)	47 (100)	1 (20)	** < 0.001**

*EMP* extensile medial parapatellar, *TTO* tibial tubercle osteotomy

Bold value indicates statistically significant *p*-values < 0.05

Regarding microbiological results, *coagulase-negative Staphylococcus* strains were the most frequent pathogens in our series, with isolates in 34.6% of cases, followed by *Propionibacterium Acnes* (17,3%) and *S. Aureus* (9,6%). Further information on PJI-causing microorganisms can be found in Table 2.

In 21 cases (40.4%), a repeated sequential TTO was performed in the first and second stage (Group A), making it the most frequently selected option in this series. In 18 patients (34.6%) a single osteotomy was performed (Group B), predominantly during the second stage (14 cases). In the remaining 13 patients (25%), the selected approach was an EMP approach, without TTO (Group C) (Table 3). Differences between surgeons with respect to the approach chosen were analysed, with a *p* value of 0.114 (no statistically significant differences). Regarding TTO-related complications, we found no cases of non-union, tibial fracture, TTO fracture or TTO displacement. In cases with repeated sequential TTO, osteotomy healing was uneventful in both stages of treatment. Interestingly, all patients with skin necrosis after the first stage (9.6%; 5/52) had been treated with an EMP approach (*p* value 0.052).

**Table 3:** Infection eradication results based on TTO timing

Groups	All patients *N* = 52 (100%)	Success *N* = 47 (90.4%)	Failure *N* = 5 (9.6%)	*p*
A–2 osteotomies	21 (40.4)	20 (95.2)	1 (4.8)	0.28
B–1 osteotomy	18 (34.6)	17 (94.4)	1 (5.6)	
C–0 osteotomies	13 (25)	10 (76.9)	3 (23.1)	

**Fig. 5:**
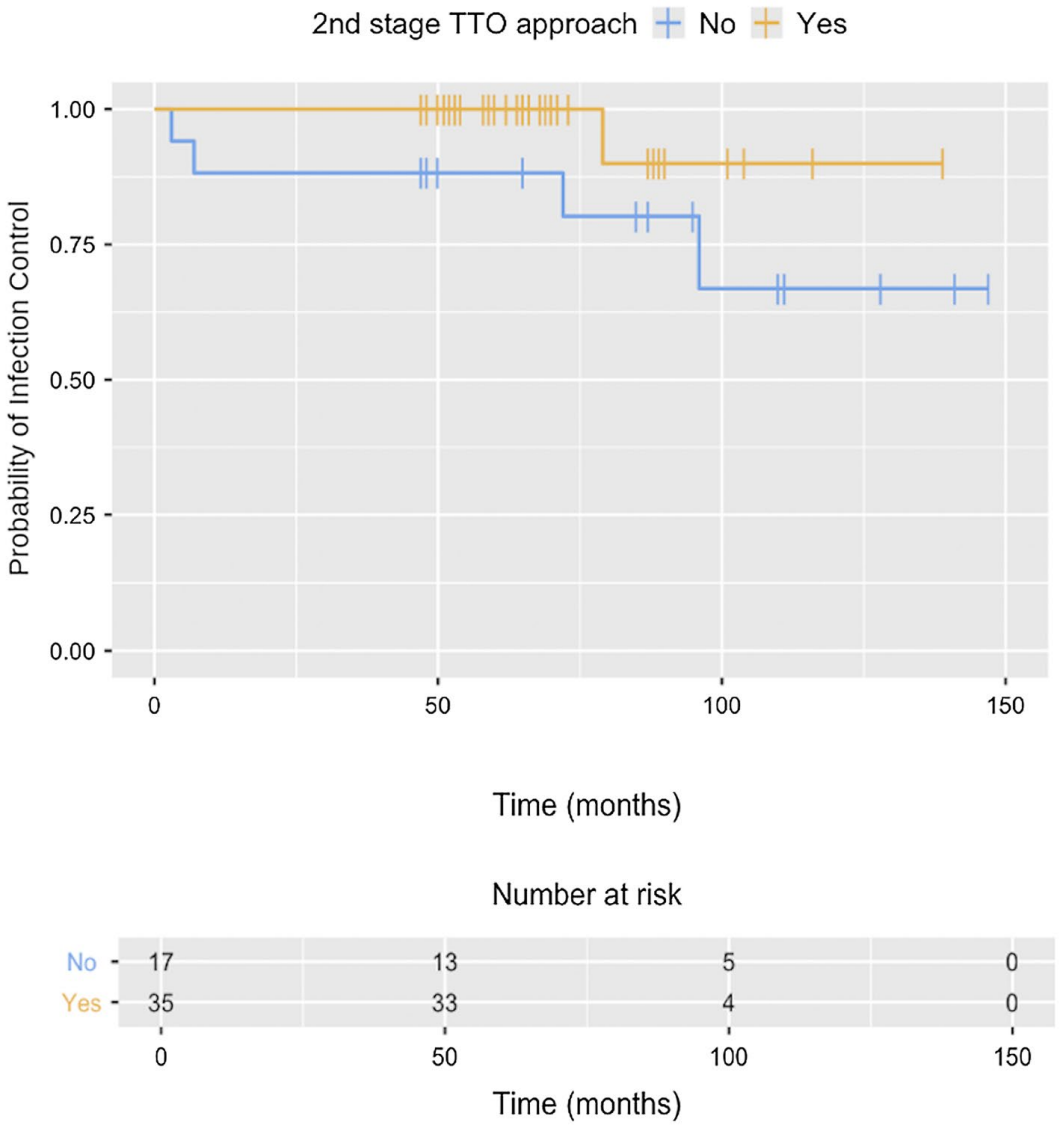
Kaplan–Meier curve. Probability of infection control depending on approach during second stage

As principal end-point of our study, after a mean follow-up of 46.2 months (range, 13.0–113.5 months), the overall infection control rate was 90.4% (47/52), following our stringent infection control criteria. On univariate analysis, a TTO performed during the second stage was associated with decreased risk of treatment failure (97.1% vs. 76.5% success rate; *p* value 0.034) (Table 2). A Kaplan–Meier curve comparing the two types of approaches during the second stage was conducted, in order to estimate the outcome of cases with short follow-up. As showed in Fig. 5, 90% of cases approached by a TTO during the second stage are likely to remain without recurrence of infection at 10 years’ time, versus 66.9% of cases approached by EMP (*p* value 0.04).

Overall, 95.2% of the patients who received repeated sequential TTO (Group A) were free of infection at the end of follow-up, compared to 76.9% success when a TTO was not performed (Group C) (*p* value 0.28). We observed that groups A and B had a similar infection control rate (95.2% and 94.4%, respectively). In both cases, the rate was clearly higher than group C (76.9%), but the difference was not statistically significant (*p* value 0.28) (Table 3).

## Discussion

In this series of chronic knee PJIs managed with a two-stage strategy employing a single CMRH prosthesis during the second stage, we found an overall infection control rate of 90.4% (47/52) after a mean follow-up of 46.2 months. Sequential repeated TTO has been proven superior in this scenario, showing a tendency toward better infection control (95.2% vs 76.9% success rate; *p value* 0.28). We identified a TTO approach in the second stage as a factor for better outcomes (97.1% vs 76.5%; *p value*: 0.034).

TTO is a well-established technique for optimizing joint visualization and protecting the extensor mechanism. It has yielded favourable clinical results in most published series [9, 23, 24]. In a septic scenario, TTO allows a better approach to the lateral and posterior areas of the knee, which are otherwise difficult to access. In addition, a properly performed TTO allows maintenance of lateral soft tissues and vascular supply to the osteotomized fragment, and is less traumatic to the surrounding soft tissues [12]. The result of such a tissue-friendly approach is demonstrated by our data, in which the skin necrosis rate is significantly higher (9.6% vs 0%; *p value* 0.052) among cases approached with a standard EMP as compared to TTO.

There is paucity of data regarding the results of TTO in the setting of infected TKA [13, 23–25]. A classical criticism of the technique in a septic scenario is the concern that it may lead to higher rates of complications including bony non-union, TTO-fragment fracture or proximal migration than are reported in a non-infected scenario. According to our data, TTO in a septic TKA is safe and reproducible. It is noteworthy that in our series no TTO-related complications were found, and the union rate at final follow-up was 100%—regardless of the number of TTOs performed during the process. The risk of complications associated with sequential repeated TTO during two-stage revision due to infection has been poorly studied [9, 12, 24]. To the best of our knowledge, the only investigation specifically addressing the performance of sequential repeated osteotomy in the septic arena is the series of 13 patients reported by Choi et al.[26]. In that series the authors found radiographically confirmed bony union in all cases. Proximal migration occurred in three of their cases; a partial proximal avulsion fracture of the osteotomy segment occurred in one case, following the second stage.

As primary end point in our series of chronic knee PJIs managed with a two-stage strategy using a CMRH prosthesis, we found an overall infection control rate of 90.4% (47/52 patients) after a mean follow-up of 46.2 months. This is especially significant when one considers the average complexity of our cases (61.5% ASA III, 50% McPherson Type B). The influence of TTO on infection control rates has been but rarely reported in the literature [27].

The rationale for this approach is based on the belief that TTO allows superior access to the infected knee, permitting much more adequate debridement. This is especially important in complex cases with multiple previous failed interventions, where stiffness and scar tissue make proper access to the joint arduous. One of the few investigations on the subject is a prospective study by Bruni D et al.[27], in which they investigated the reinfection rate in knee PJI patients treated with two-stage exchange arthroplasty using either a TTO or a quadriceps snip (QS) for exposure at the time of reimplantation. In their series, they found no difference in reinfection rates between groups. As a criticism, it should be noted that they mentioned no use of validated criteria for success. Conversely, our results support this idea: performing a TTO in the second stage (sequential or isolated) was identified as a factor for superior infection control outcomes (97.1% vs. 76.5%; *p value* 0.034). However, our mean follow-up was significantly shorter than that of Bruni D et al. [27]; 46.2 months versus 144 months, respectively. To amend that to some extent, we analysed the probability of remaining without recurrence of infection with a Kaplan–Meier estimate **(**Fig. 5**)**, finding that the infection control rate at 10 years’ time would likely be 90% for cases approached by a TTO in the second stage, versus 66.9% in patients approached by EMP (*p value* 0.04).

The current study did not find a statistically significant difference in relapse rates between the group using sequential repeated TTO (Group A) and the non-osteotomy group (Group C). The incidence of infection control (76.9% for the non-osteotomy group and 95.2% for the sequential repeated TTO group), limits powering such a study. However, the 18.3% improvement in infection control suggests that use of sequential repeated TTO merits further study.

We recognize the limitations of our study. Designed as a retrospective non-randomized analysis, all data was gathered from medical records; being the inability to obtain all relevant information one of the drawbacks of our study. It is a single-institution study, hence limiting the generalization of our results. Three different surgeons operated and treated the study’s patients, which increases variability; however, all of them followed the same pre-established protocol, assisted each other in performing the procedures, and are specialised in treating musculoskeletal infections. In addition, differences between surgeons in terms of approach chosen were assessed without finding statistically significant differences. Nevertheless, the selected approach in each specific case could establish a bias. It would be logical to think that osteotomy has been chosen in the most complex cases (selection bias), but the fact of obtaining better results in TTO cases validates the usefulness of this approach in complex cases of infection. Other limitations that should be considered are the sample size and follow-up period. However, both these parameters were comparable or superior to previously published studies. Although the limited sample size impeded a solid multivariable regression analysis, in the best of our knowledge this is the largest series addressing the performance of sequential repeated osteotomy in the septic arena. Because of these limitations, our results should be interpreted with caution; studies with a longer follow-up period and larger patient bases are needed.

## Conclusions

The results of the current study suggest that a TTO (repeated or isolated) in a two-stage exchange strategy is a reasonable option and offers a high rate of infection control and low complication rate in complex cases of knee PJI.

## Data Availability

The data that support the findings of this study are available from the corresponding author (Marta Pérez), upon reasonable request.
